# Personalized Dose Selection for Treatment of Patients with Neuropsychiatric Disorders Using tDCS

**DOI:** 10.3390/brainsci14121162

**Published:** 2024-11-21

**Authors:** Sagarika Bhattacharjee, Rajan Kashyap, Vanteemar S. Sreeraj, Palanimuthu T. Sivakumar, Ganesan Venkatasubramanian, John E. Desmond, S. H. Annabel Chen, T. N. Sathyaprabha, Kaviraja Udupa

**Affiliations:** 1 Department of Neurophysiology, National Institute of Mental Health & Neurosciences NIMHANS, Bengaluru 560029, Indiakaviudupa@gmail.com (K.U.); 2 Department of Neuroimaging and Interventional Radiology, National Institute of Mental Health & Neurosciences NIMHANS, Bengaluru 560029, India; rajankashyap6@gmail.com; 3 Department of Psychiatry, National Institute of Mental Health & Neurosciences NIMHANS, Bengaluru 560029, India; vs8sreeraj@yahoo.com (V.S.S.); pts@nimhans.ac.in (P.T.S.); venkat.nimhans@gmail.com (G.V.); 4 Department of Neurology, School of Medicine, The Johns Hopkins University School of Medicine, Baltimore, MD 21218, USA; jdesmon2@jhmi.edu; 5 Department of Psychology, Nanyang Technological University, Singapore 639798, Singapore; annabelchen@ntu.edu.sg; 6 Centre for Research and Development in Learning (CRADLE), Nanyang Technological University, Singapore 639798, Singapore; 7 Lee Kong Chian School of Medicine (LKC Medicine), Nanyang Technological University, Singapore 639798, Singapore

**Keywords:** individualization, atrophy, precision medicine, transcranial direct current stimulation, HD—conventional montage trade-off

## Abstract

**Background**: Individualizing transcranial direct current stimulation (tDCS) parameters can improve precision in neuropsychiatric disorders. One important decision for the clinician is the selection of an appropriate montage—conventional or high-definition (HD)—to implement dose-controlled tDCS while maintaining the patient’s safety. **Method**: The present study simulated tDCS administration using T1-weighted brain images of 50 dementia, 25 depression patients, and 25 healthy individuals for two conventional and HD montages, targeting the regions of interest (ROIs) in the dorsal and ventral pathways that support language processing. For each tDCS configuration, the electric fields at the ROIs and the individualized dose required to achieve the desired current intensity at the target ROI across the subjects were estimated. Linear regression was performed on these parameters. **Result**: A significant relationship between atrophy and current dose that varies according to the disease was found. The dementia patients with significant brain atrophy required a higher personalized dosage for HD montage, as the current intensity at the target ROIs was lower and more variable than that of conventional montage. For dementia, tDCS individualization is pathway-dependent, wherein HD configuration of the dorsal route requires current dosages above the safety limit (>4 mA) for 46% of individuals. However, there was no significant difference in electrode configurations between the HD and traditional setups for depression and healthy volunteers without significant brain atrophy. **Conclusions**: HD-tDCS with fixed locations is limited, making conventional tDCS more effective for dose-controlled applications. In patients with atrophy, individualized adjustments based on simulations are needed due to the variable stimulation strength in the ROI.

## 1. Introduction

Noninvasive brain stimulation, like transcranial direct current stimulation (tDCS), is a prominent treatment approach extensively employed in neuropsychiatric populations [1,2]. Its growing popularity is attributed to its clinical effectiveness in managing treatment-resistant symptoms, simplicity of application, cost-effectiveness and lower incidence of adverse effects [3]. The tDCS uses a low electrical current to influence neural activity in cortical networks. The generalizability of its therapeutic potential is limited due to inconsistent effects [4,5]. Studies have suggested that the variability of tDCS’s effects is due to variability in the current intensity (electric field) at the brain’s targeted region of interest (ROI) [6]. This happens because the conventional practice is to apply a fixed intensity of tDCS current dose (e.g., 1 or 2 mA) uniformly across the individuals of a group. This one-size-fits-all strategy has led to significant variability in the electric fields in the ROIs due to inter-individual anatomical variability in cerebrospinal fluid, gray matter, white matter volumes, skull conductivity, etc. [7].

Recent studies have suggested that the inter-individual variability in tDCS could be reduced by alternating the current dose across the subjects in a group to ensure that all subjects receive the same electric field strength in the ROI [6]. For this approach, current flow modeling, which estimates the electric fields in an individual’s brain based on the placement of the tDCS electrode and current dose, would be adopted. The qualitative agreement between the calculated and measured electric fields has been established [8,9,10]. Evan et al. [6] performed electric field modeling of T1-weighted structural MRIs of 50 healthy adults (aged 22–35, 23 males, 27 females) using the conventional montage (CP5-FC1) and calculated the individualized tDCS current doses required to achieve a consistent intensity at M1 (the target ROI) for each participant. They demonstrated that individualizing the dose reduced the variability of the current distribution in the ROIs compared to the conventional fixed-dose approach.

It has been suggested that personalizing tDCS doses in clinical settings can enhance patient outcomes [11], though factors such as aligning the task with the targeted brain areas are also crucial [12]. While dose-controlled tDCS administration might appear logical, clinicians need more clarity before adopting such an approach in a patient population. First, it is essential to investigate whether individualized current doses will be within the safe range (i.e., within 0–4 mA) [13]. While doing so, it is crucial to consider neuropsychiatric disorders with and without cortical atrophy since the current distribution in the ROIs for the two categories of patients may differ owing to the differences in brain anatomy [14] and tissue type distribution [7,15]. Second, it is crucial to determine the montage configuration—high-definition (HD, 4 × 1) or conventional (1 × 1)—that delivers the dose-controlled tDCS. This is important because, just as individual anatomy influences the distribution of electric fields, so does the choice of tDCS montage [9]. Additionally, HD-tDCS results in enhanced stimulation focality despite utilizing the same current dose (1–2 mA) as conventional montages [16]. However, increased focality may introduce more significant uncertainty in group-level outcomes because E-fields become more influenced by local anatomical variability in individuals [17].

In this cross-sectional study, we will assess the suitability of individualizing tDCS doses by simulating electric fields from T1-weighted images in two neuropsychiatric groups—patients with dementia and those with depression—compared to healthy controls. These groups were selected because dementia involves significant brain atrophy [18,19,20], whereas depression typically does not [21]. We will use electric field modeling to determine personalized dosages for two conventional and two HD montages previously used to target the dominant left hemisphere’s dorsal and ventral language pathways [22,23,24]. Our goal is to provide a pipeline to help clinicians make informed decisions regarding dose selection when personalizing tDCS treatment for neuropsychiatric disorders. 

## 2. Methodology

### 2.1. Data Description

Strict criteria were maintained for subject selection. Demographic constraints were maintained by confining our selection of brain images to the patient/healthy population of India. Consequently, studies previously conducted in India and that are ongoing at our institute were considered. Fifty dementia patients met the inclusion and exclusion criteria: age above 50 years, clinically diagnosed dementia case with HMSE ≤ 23 [25], and no history of depression or any other neuropsychiatric disorders according to publicly available data under the Longitudinal Aging Study in India-Diagnostic Assessment of Dementia (LASI-DAD) [26,27] collected at the National Institute of Mental Health and Neuro Sciences (NIMHANS), Bangalore, India. T1 weighted images of these patients were obtained using a 32-channel Philips Ingenia 3T scanner with TR = 2300 ms, TE = 2.03 ms, and voxel size = 1 × 1 × 1 mm^3^. Details about the LASI-DAD study protocol can be found in Jinkook et al. [27]. Twenty-five T1-weighted images for depression patients and age-sex-matched healthy volunteers were obtained from an ongoing study in NIMHANS. For the depression images, we selected individuals who matched the inclusion criteria and were diagnosed with moderate to severe depression cases (HDRS score ≥ 14) and had no history of cognitive impairments. The healthy volunteers did not have any history of cognitive impairments or any neuropsychiatric disorders. The T1-weighted single-shot three-dimensional (3D) turbo field echo (TFE) image was acquired on a 3T Philips Ingenia CX machine using a 32-channel phased-array coil at 1 × 1 × 1 mm voxel size, with slice thickness = 6 mm, TR = 5000 ms, TE = 10 ms, flip angle = 90°. The present study was approved by the institutional ethical review committee of National Institute of mental Health and neurosciences approved on 22 December 2023 with approval number: NIMHANS/39th IEC (BS & NS DIY.)/2023.

### 2.2. Electric Field Simulation

**Figure 1:**
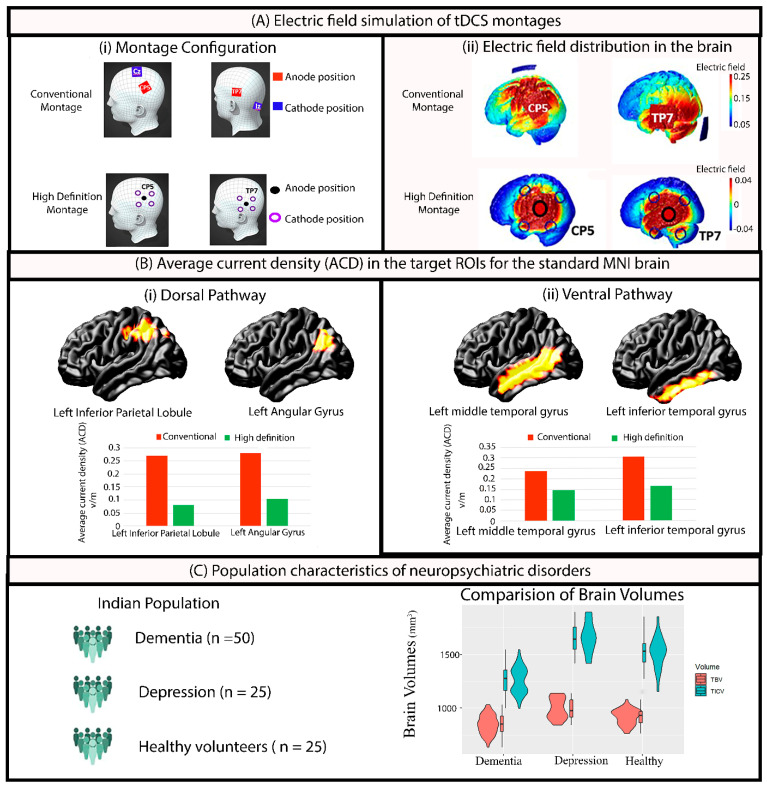
(**A**) Electric field simulation of tDCS dorsal and ventral pathway montages for both conventional and high-definition (HD) configurations demonstrating the (**i**) electrode positions, and (**ii**) electric field distributions across the brain regions. (**B**) Average current density (ACD) showing the electric field strength on the standard MNI brain (used as a reference for the calibration of doses) for conventional and HD configurations across (**i**) the dorsal pathway with two target ROIs: left inferior parietal lobule and left angular gyrus; and (**ii**) the ventral pathway with two target ROIs: left middle temporal gyrus and left inferior temporal gyrus. (**C**) Brain volumetric characteristics of the three groups (dementia, depression, and healthy) highlighting differences in the TBV [Total brain volume (GM + WM)] and TICV (Total intracranial volume (TBV + CSF)).

In our previous studies [22,23], we showed that montages with (1) anode at CP5 and a cathode at CZ can target the inferior parietal lobule in the dorsal pathway, and (2) an anode at TP7 and a cathode at the nape of the neck can appropriately target the left middle/inferior temporal lobule in the ventral pathway. For the HD-tDCS simulation, four surrounding cathodes with a central anode were used at (i) CP5 for the dorsal pathway and (ii) TP7 for the ventral pathway. While the conventional electrode size was 5 × 5 cm^2^ for both CP5_CZ and TP7_Nape of the neck, the HD configuration (4 × 1) adopted ring electrodes of 1 cm radius (Figure 1A(i)). For each MRI, simulations were performed for the standard 2 mA current dose, totaling 400 simulations in ROAST [28] (100 MRIs × 4 montage configurations = 400). Default conductivity values for tissues (white matter: 0.126 S/m, gray matter: 0.276 S/m, cerebrospinal fluid: 1.65 S/m, bone: 0.01 S/m, skin: 0.465 S/m, air: 2.5 × 10^−14^ S/m, gel: 0.3 S/m, electrode: 5.9 × 10^7^ S/m) were applied during the simulation. The ROAST simulation provided the x, y, and z coordinates of brain regions and the current density (mA/m^2^) value at each location in the native space (Figure 1A(ii)).

For each simulated montage in ROAST, i-SATA (MNI) [29,30] identifies the location (x, y, and z coordinates) of all points in the cortex in the MNI-152 template and maps these points to the Automated Anatomical Labeling (AAL) atlas [31] with 116 cortical and subcortical areas. The current density magnitude corresponding to each location (x, y, and z) is then used to calculate the following measures. 

#### 2.2.1. Average Current Density (ACD)

The average magnitude of current density (ACD) received by each cortical ROI of the brain is calculated by averaging the current density magnitudes corresponding to all the x, y, and z coordinates (voxel) within the defined ROI (Figure 1C). Thus, the ACD was estimated for the target ROI in the dorsal pathway: Left_Inferior_Parietal_Lobule_ACD. Three other nearby ROIs (Angular_Gyrus, Supramarginal_Gyrus, and Superior_Temporal_Gyrus) were also considered to be in the dorsal pathway because of their auxiliary contribution to language processing. The ACDs are denoted as Left_Angular_Gyrus_ACD, Left_Supramarginal_Gyrus_ACD, and Left_Superior_Temporal_Gyrus_ACD [32]. Similarly, the ACDs for the two ROIs estimated for the ventral pathway are denoted as Left_Inferior_Temporal_Lobule_ACD and Left_Middle_Temporal_Lobule_ACD, respectively.

#### 2.2.2. Individualized Dosage

The ACDs obtained at the desired 6 ROIs following the simulation of the four montages (mentioned in Section 2.2) on standard MNI images using a fixed dosage of 2 mA were set as the target intensities ($M_{Reference-Intensity}$), intended to be achieved and maintained consistently across all individuals. Such targets were fixed because 2 mA was the highest intensity used in most prior studies that showed physiological responses following tDCS [33,34,35,36] and MNI is a standard brain template [37]. $M_{Actual-Intensity}$ is the intensity at the same target ROI obtained after the simulated fixed dose of 2 mA on individual images. The individualized dosage was calculated for each ROI separately. This required using the following formulae introduced by Evans et al. [6]. The required individualized dosage for ROIs in the dorsal pathway are denoted as Left_Angular_Gyrus_Dose, Left_Supramarginal_Gyrus_Dose, and Left_Superior_Temporal_Gyrus_Dose [32]. Similarly, the dose for the two ROIs estimated for the ventral pathway are denoted as Left_Inferior_Temporal_Lobule_Dose and Left_Middle_Temporal_Lobule_Dose, respectively.

$${Individualised}_{Dose}=\frac{M_{Reference-Intensity}}{M_{Actual-Intensity}}\times {Fixed}_{Dose}\;,\;where\;{Fixed}_{Dose}=2\;mA$$

#### 2.2.3. Dose Target Determination Index (DTDI)

The i-SATA (MNI) output, encompassing the average current density in the target region of interest (ROI) and non-target areas, serves as the basis for calculating the Dose-Targeted Density Index (DTDI) for a simulated montage at a given current dose. In this process, we identify the ROI with the maximum average current density (peak region) among all ROIs. The *DTDI* is then computed as follows, as introduced by [29]

$$DTDI=\frac{Average\;Current\;Density\;at\;the\;Target\;ROI}{Maximum\;value\;of\;average\;current\;density\;formed\;at\;any\;ROI}$$

*DTDI* values range from 0 to 1. An ideal tDCS setup expects the maximum stimulation intensity (ACD) to be received at the target ROI, resulting in a *DTDI* value of 1. However, the peak intensity can be received at a non-targeted ROI. For an ROI, DTDI determines the probability of hitting the target ROI with peak current intensity.

### 2.3. Estimation of Volume Parameters

The volume parameters of each T1 image were computed using the CAT12 toolbox (version 12.7) [38]. Correcting for bias-field inhomogeneity and spatial normalization through the DARTEL algorithm, the images underwent segmentation to obtain the total volume of gray matter (GM), white matter (WM), and cerebrospinal fluid (CSF). Modulating segmented images involved multiplying tissue class images aligned with the template by the Jacobian determinant derived from spatial normalization [38,39]. This step corrected for individual differences in brain size [40]. Subsequently, the segmented images underwent smoothing by convolving with an isotropic Gaussian kernel of 8-mm FWHM size [38]. The total brain volume was calculated as the sum of the GM and WM volumes. The total intracranial volume was calculated as the sum of the TBV and CSF volumes. The global brain atrophy was calculated as the ratio of TBV and TICV [41], denoted as

$$\frac{Total\;Brain\;Volume\;\left(GM+WM\right)}{Total\;Intracranial\;volume\;(GM+WM+CSF)}$$

### 2.4. Statistical Analyses

The atrophy differences between the three groups were evaluated using one-way ANOVA. To understand the relation between the simulated E-field values and montage types for each patient group, separate linear regressions were performed using ACD, personalized dose, and DTDI values at each target ROI as dependent factors and montage type (conventional and HD) and group (dementia, depression and healthy) as independent factors. When an analysis at each ROI was performed, the current distribution at other ROIs was not considered, because each montage (dorsal/ventral) was simulated with the intention of targeting the individual ROI. Similarly, linear regressions were performed to understand the relation between atrophy and personalized dosages at the ROIs, with personalized dose at target ROI as the dependent factor and atrophy as the independent factor. For an ROI that was found significant, the patient group (dementia, depression, or healthy) was introduced as a cofactor in the regression analysis. Post hoc comparison was performed for estimated marginal means using Tukey’s method to control for multiple comparisons.

## 3. Results

### 3.1. Demographics and Clinical Results

The patients were evaluated for depression using the Hamilton Depression Rating Scale (HDRS) [42] and cognition was measured using the Hindi version of the Mini-Mental State Examination (HMSE) [43]. The clinical details of the patient groups are provided in Table 1.

### 3.2. Volumetric Results

Both the TBV and TICV significantly (^TBV^ β_1_ = −150.5, SE = 15.9, df =197, t = −9.48, *p* < 0.0001, ^TICV^ β_1_ = −401, SE = 23.3, df = 197, t = −17.22, *p* < 0.0001) decreased in dementia patients (TVB: 843 ± 9.16, TICV: 1261 ± 13.4) compared to those with depression (TVB: 993 ± 12.96, TICV: 1662 ± 19.0). A similar trend between dementia and healthy volunteers [(TVB: 927 ± 12.96, TICV: 1529 ± 19.0)] was also observed [(^TBV^ β_1_ = −84.5, SE = 15.9, df = 197, t = −5.3, *p* < 0.0001), and (^TICV^ β_1_ = −268, SE = 26.9, df = 197, t = −11.51, *p* < 0.0001)]. Differences were not significant between the depression and healthy group (Figure 1C). This indicated that atrophy was significant only in patients with dementia. One-way ANOVA confirmed this by showing significant differences (f = 78.65, *p* < 2 × 10^−16^ ***) between the three groups in the atrophy. Post hoc comparison showed the dementia group to be significantly (β_1_ = 0.07, SE = 0.006, df =197, t = −11.02, *p* < 0.0001) different compared to the depression and healthy group (β_1_ =0.06, SE = 0.006, df = 197, t = 9.31, and *p* < 0.0001). As expected, healthy volunteers were not significantly different from the depression group (β_1_ = −0.01, SE = 0.007, df =197, t = −1.47, *p* = 0.3).

### 3.3. Electric Field Simulation Results

**Figure 2:**
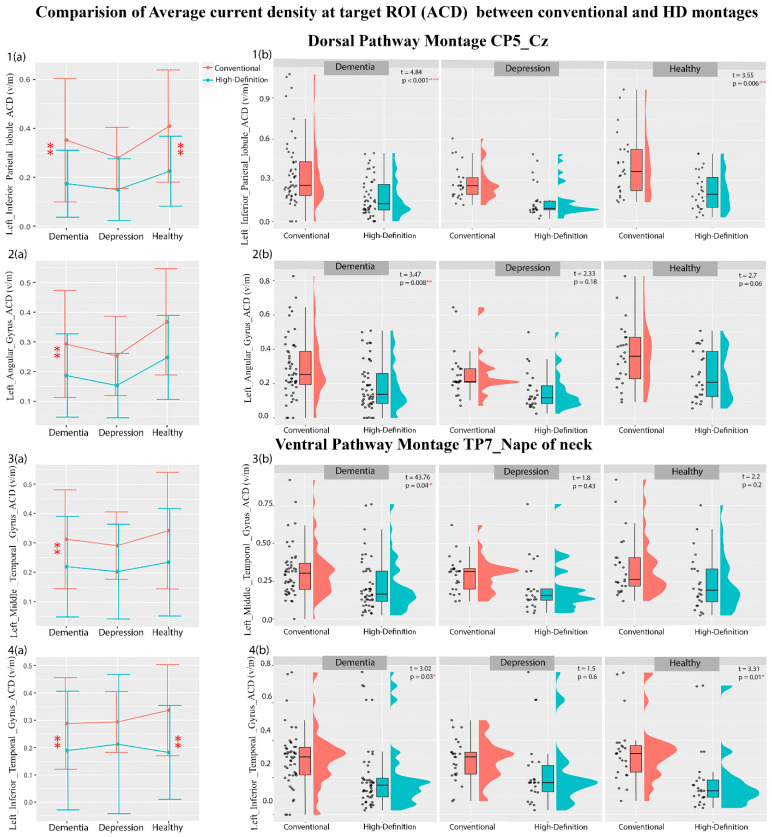
The plot of mean ± standard deviations of ACD (**a**(**1**)–**a**(**4**)) highlighting their distribution across the subjects (**b**(**1**)–**b**(**4**)) for dorsal and ventral ROIs using conventional and high-definition montages for dementia, depression, and healthy volunteers. Level of significance denoted by * <0.05. ** <0.01, *** <0.001.

**Figure 3:**
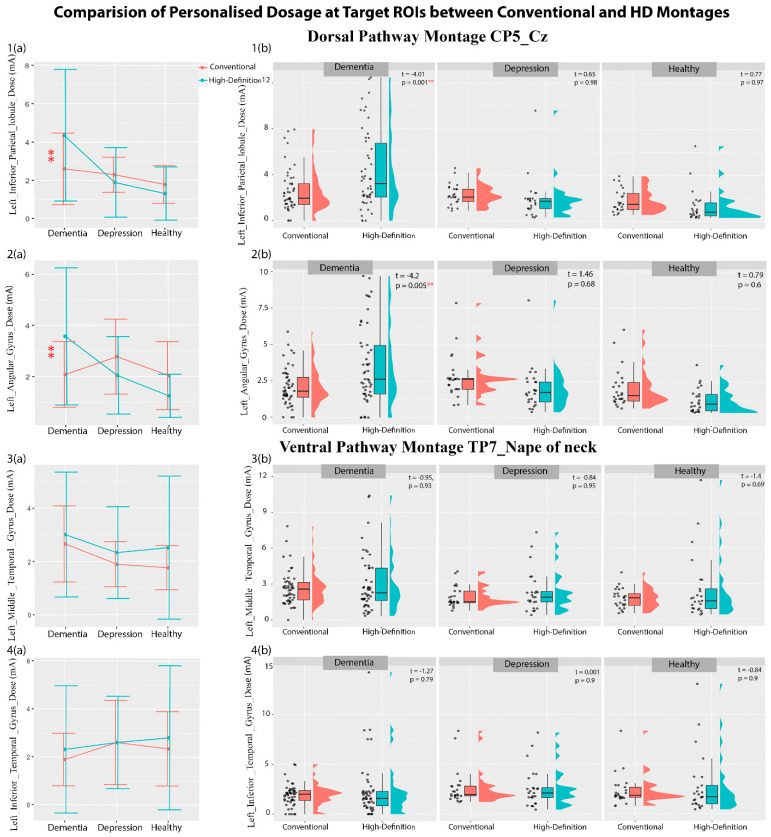
The plot of mean ± standard deviations of personalized dosages (**a**(**1**)–**a**(**4**)) highlighting their distribution across the subjects (**b**(**1**)–**b**(**4**)) for dorsal and ventral ROIs using conventional and high-definition montages for dementia, depression, and healthy volunteers. Level of significance denoted by ** <0.01.

Regression analyses with ACD, personalized dose, and DTDI values at each target ROI of the dorsal and ventral pathway as dependent factors and patient group (dementia, depression, and healthy volunteer) and montage type (conventional and HD) as independent factors were carried out. The analysis showed a significant main effect of montage type and an interaction effect of montage type and patient group (Table 2). The mean and variance in Table 2 show that the ACD of a conventional montage is higher than that of an HD montage, whereas the dose for an HD montage is higher than that of a conventional montage for both dorsal and ventral ROIs. However, the F-test of variance shows that an HD montage has a higher variability of ACD and dosage than a conventional montage. The post hoc comparison (Figure 2 and Table S1 of the Supplementary Materials) shows that the conventional montage delivered significantly higher current intensity than the HD electrodes at the left inferior parietal lobule and left angular gyrus of the dorsal pathway and the left middle temporal gyrus and left inferior temporal gyrus of the ventral pathway. These differences in the ACD were seen in the dementia patients and healthy volunteers only, not in the depression group. When the personalized dosage was calculated (Figure 3, and Table S1 in Supplementary Materials), the conventional montage required a lower dosage than the HD electrodes for the left inferior parietal lobule and left angular gyrus in the dorsal pathway in dementia patients only. No dosage difference was seen for the ventral pathway in the three groups. Interestingly, when individual data were plotted (see Figure 2 and Figure 3), we observed that some dementia patients may require a dosage well above the tolerable range of 4 mA, especially for 46% of HD montages. When the *DTDI* measures (the probability of hitting the target region with peak current) were compared, no significant difference was seen between conventional and HD montage configurations in any of the three groups (Table S1 and Figure S1 in Supplementary Materials). This suggests that conventional and HD montages have equal chances of hitting the target region with the peak current, although HD-tDCS is more focl in its current distribution.

#### Cross Validation of the Result

This cross-sectional study aimed to assess the relationship between personalized dosage at a target ROI with montage type (HD or conventional) and patient group (dementia, depression, and healthy controls) using regression analysis. A required sample size of 54 was calculated using an effect size of 0.25 (from Evans et al. [1]), 90% power, and F-statistics in G Power. However, after applying inclusion and exclusion criteria, 50 dementia patients, 25 depression patients, and 25 healthy volunteers were recruited, leading to unequal sample sizes. To ensure the effect size remained significant, the power analysis was reconducted. The results indicated that the power ranged from 80% to 95% (see Table 2 for with respective R^2^ values) with an α error probability of 0.05. For further confirmation, we repeated our analysis with 25 subjects per group. The findings did not change.

### 3.4. Relation of Atrophy and Personalized Dosage

**Figure 4:**
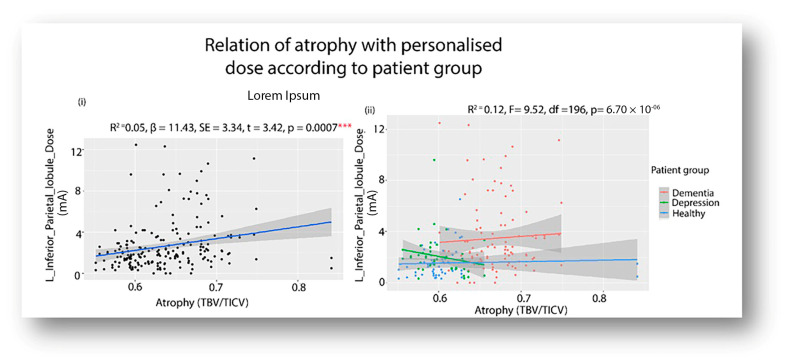
Showing (**i**) The significant (*p* < 0.001) relationship between the atrophy parameter and personalized doses at the target ROI at the left inferior parietal lobule, and (**ii**) its significant variation across the three groups: depression, dementia, and healthy volunteers. (*** denotes significance level < 0.001).

For each pathway, a regression was performed to ascertain the relationship between atrophy and personalized dose at the target ROI (Figure 4). The atrophy was only significantly related to personalized dosage at the left inferior parietal lobule (R^2^ = 0.05, β = 11.43, SE = 3.34, t = 3.42, *p* = 0.0007) (Figure 4(i)). The patient’s group was then introduced as a cofactor (Figure 4(ii)). The main effect of patient group was found to be significant for both dementia and depression (*^Depression^* β = −1.3, SE = 0.49, t = −2.6, *p* = 0.009, *^Dementia^* β = −1.8, SE = 0.46, t = −3.97, *p* = 9.94 × 10^−5^ ). Post hoc comparisons of dosage (at a mean atrophy of 0.63) found the dementia group (3.44 ± 0.263) to be significantly different (β = 1.3, SE = 0.49, df = 196, t = 2.6, *p* = 0.02) from depression (2.14 ± 0.36) and also significantly different (β = 1.86, SE = 0.47, df = 196, t = 3.97, *p* = 0.0003) from the healthy volunteers (1.571 ± 0.34).

## 4. Discussion

The present study intended to investigate the feasibility of dose-controlled tDCS on neuropsychiatric disorders by simulating the electric field using T1-weighted brain images. To this end, electric field modeling was used to calculate the current dosage of tDCS so that a constant current intensity was maintained at the target ROI across the individuals of a group. Since current tDCS dosages have been demonstrated to be safe and tolerable up to 4 mA, as reported in prior studies [13,44], emphasis was placed on determining whether the conventional or HD montage configuration would be more appropriate for adopting a dose-controlled individualized tDCS approach, prioritizing the patient’s safety (i.e., dosage < 4 mA). The study made the following key findings. (1) Generally, the conventional montage results in more current intensity and less inter-individual variability at a target ROI than the HD configuration. Therefore, conventional montages require less current dosage (within the safety limit) for personalization. (2) Depending on the pathway (target ROI) and patient group (atrophied), the HD configuration might require dosages beyond the tolerable range. For example, 46% of dementia patients needed a very high dosage (>4 mA) in the HD configuration while targeting the left inferior parietal lobule of the language dorsal pathway. The same patients did not require a high dosage (in HD configuration) when the left inferior temporal lobe in the ventral language pathway was targeted. Interestingly, such variations were not found for non-atrophied disorders (depression) and healthy individuals. Both the HD and conventional montages required current dosage within safety limits across the two pathways (dorsal and ventral) for depression and healthy volunteers. And lastly, brain atrophy was found to be significantly associated with the personalized dosages at the left inferior parietal lobule that varied across the patient groups. To our knowledge, the relationship between atrophy and personalized dosages in neuropsychiatric patients has not been reported previously. The present study’s findings emphasize the important role atrophy holds in the personalization of tDCS parameters.

The difference between conventional and HD electrodes has been discussed in several studies. For example, many studies show different physiological responses in the form of event-related potentials [45] and cortical plasticity measured by magnetic evoked potential [46] after using conventional versus HD electrodes. However, although HD configurations have high focality, the electric field strengths induced at the target ROI show very high inter-individual variability, owing to differences in brain anatomy [17,47]. Most studies that compared HD and conventional tDCS systems were conducted on healthy individuals and reported that the calculated individualized dosage remains within the safety limits of tDCS administration [48]. We also found this in our study of the healthy group.

However, the findings in the clinical population may differ depending on the disorder (with atrophy) and the ROI to be stimulated. To a certain extent, this pattern was also reported in the work of Mizutani-Tiebel et al., [49], where a significant difference in the electric field strength was observed between healthy volunteers and patients with major depressive disorder and schizophrenia.

A recent study using the datasets of 240 healthy individuals demonstrated that when the electrode position for a tDCS montage is not precise, the current dose at the target region is reduced by 29–43% [50]. Such deviations are higher for HD montages than conventional montages. The researchers specifically recommended neural navigation to minimize electrode positioning drift and reduce inter-individual variability, especially for the HD configuration. The present study supports their findings and reports for the first time that the required individualized dosage may not be within tolerable limits for dementia patients, especially for an HD configuration targeting the dorsal language pathway.

With that said, researchers are trying to advance the field of precision medicine, and a few other individualized dose-control methods that could reduce variations in tDCS electric field strength at the target ROI have been introduced recently. For example, one approach involves individualization using the motor threshold induced by transcranial magnetic stimulation (TMS) [51]. Another method considers head circumference as an indirect measure of an electric field to achieve a consistent current density at the target ROI across individuals [52]. These innovative methods were developed on healthy individuals and presumes a linear relationship between motor threshold or head circumference and current density at the target ROI. However, the in vivo validation of such assumptions remains to be conducted, and their manifestation in the clinical population is pending. The electric field-based simulation approach delineated by Evans et al. [6] requires MRI scanning and thus might be expensive. However, in a clinical population where precision, safety, and comfort are priorities, we suggest that tDCS-based clinical trials should perform prior adjustments of the stimulation strength based on patient-specific simulations.

Furthermore, tDCS electrode montages can also be personalized by modifying the placement, number, shape, and size of the electrodes, often using MRI-based computational models to tailor the current flow for each participant. Neuronavigation tools and M/EEG data can further refine electrode placement, especially when targeting specific neural activities like epileptogenic regions. Recent multisession tDCS studies [53] using individualized montages have shown promising improvements in conditions such as Alzheimer’s disease, major depressive disorder, and epilepsy, although some epilepsy studies lacked control conditions. Despite these advancements, there remains considerable variability in individual responses, suggesting that simply customizing electrode montages may not fully address this issue [53].

Moreover, the positive relationship between brain atrophy and current density has been demonstrated in prior studies on healthy aging brains [7,54]. They found that less current is needed to reach brain tissues with more atrophy due to the critical role of CSF in delivery of the current [14,55,56]. Since CSF is a better conductor than brain tissue, electrical current is “shunted” around the CSF, resulting in less current entering brain areas [15]. The authors of [7] also reported that adjusting the current dose in the aging brain may be necessary to ensure that a sufficient amount of current reaches the target brain regions. Individuals with more atrophy are likely to require more current than those with less atrophy. Therefore, the practical application of tDCS may need to take into account the degree of brain tissue loss (atrophy) when determining the personalized current dose in neuropsychiatric patients.

Currently, personalization efforts focus on maximizing the current delivered to the target region of interest, which is critical for effective tDCS responses. However, other key factors, such as the strength [6] and orientation [57] of the electric field, are often not individualized. The present study emphasizes adjustments of the stimulation strength based on patient-specific simulations with a priori fixed positions. Future studies should investigate these additional current flow features to determine their role in optimizing stimulation outcomes. Additionally, factors such as brain health and functional state need to be considered for fine-tuning stimulation protocols. Though computationally and methodologically demanding, this comprehensive approach is essential to developing more reliable and accessible tDCS methods capable of consistently inducing desired neurophysiological and behavioral changes.

## 5. Limitation and Future Direction

There are a few limitations in the study that warrant careful interpretation of the results.

First, it is known that focality is highest with HD electrodes [17] and that the degree of focality increases with a decrease in ring radius [16]. Moreover, many experimental studies have shown that HD-tDCS produces better neurophysiological responses than conventional electrodes in healthy adults [9,58,59]. However, little work has been done regarding atrophy’s effects on the individualized dosing of tDCS. This is particularly relevant at the clinical level, since patients with atrophy affecting specific brain areas could benefit differently from HD/conventional montages in relation to the target site for stimulation. As tDCS vitally needs precision dosing strategies due to the inherent anatomical variability across individuals, this simulation demonstrates a simple and coherent pipeline for estimating the tDCS current dose. Nevertheless, future empirical studies are required to assess the impact of individualized dosage on behavioral outcomes.

Second, the required dosage was based on a 2 mA template model for individual adjustments. We recognize that the exact threshold for a physiological response in the target ROI of dementia patients is unknown, as less than 45% of participants showed the expected increase in corticospinal excitability after anodal tDCS of 2 mA [60]. Due to this uncertainty, we chose 2 mA as a somewhat arbitrary baseline. While this is the upper limit observed in most studies [53], responses have also been noted at lower doses such as 1 or 1.5 mA. This serves as a preliminary standard, with further research, including animal studies, needed to determine the precise threshold for dementia patients.

Third, the language network was chosen for tDCS simulation because it covers a broad brain region with two processing routes (dorsal and ventral), which we have previously shown to be modulated using our chosen set of montages. Although dementia and depression are not primarily associated with linguistic deficits, the present study explored the question, “Given any target region, which personalized strategy should a clinician adopt to stimulate the underlying neural network?”. The appropriate answer to this question depends on the type of patient (atrophied or non-atrophied) the clinician is handling. The present study serves as a roadmap to provide insights into the trade-offs between montage configuration, atrophy, dose, and the likelihood of targeting the correct region with the peak current intensity. Notably, montage-related dose differences were observed only in the dorsal pathways of dementia patients, suggesting that the effect is target-region and patient-type dependent. Future studies should conduct similar analyses on other regions, such as the left prefrontal cortex, to ascertain the personalized dose strategies in tDCS. Another arena that we did not consider was that of stroke patients, in whom the lesioned brain distorts the current distribution of tDCS; this topic is as yet underdeveloped. This study encourages personalized tDCS research to be undertaken on the spectrum of neuropsychiatric disorders.

## 6. Conclusions

This study shows that HD montages require a higher personalized dosage in dementia patients, as the current reaching target ROIs is lower than when conventional tDCS is used. In contrast, no significant differences in current delivery were found between conventional and HD configurations in depression or healthy individuals without atrophy. For dementia patients, conventional tDCS, which covers a larger brain area, appears safer than focal HD-tDCS with fixed positions when dose-controlled tDCS is planned.

To conclude: (i) In patients with atrophy, the stimulation strength in the ROI varies due to brain volume loss, requiring individualized adjustments based on patient-specific simulations. (ii) HD-tDCS with fixed locations is limited by its focal nature and lack of consideration for an individual’s brain structure, making conventional tDCS more effective in individualized dose-controlled tDCS. If only fixed-location tDCS is possible, conventional tDCS is preferable. Ideally, individualized montages based on head models should be used to optimize stimulation strength and location.

## Figures and Tables

**Table 1:** The clinical demographics of the patients.

Group	Age (Sex)	HMSE Score	HDRS Score
Dementia (n = 50)	66.38 ± 6.04, (20F, 30M)	22.6 ± 5.5	9.98 ± 5.39
Depression (n = 25)	67.52 ± 6.03 (10F, 15M)	27.66 ± 2.55	19.2 ± 7.105
Healthy (n = 25)	65.85 ± 5.82 (11F, 14M)	29.40 ± 2. 32	1.9 ± 0.63

HDRS (Hamilton Depression Rating Scale), HMSE = Hindi version of Mini-Mental State Examination, F = Female, M = Male.

**Table 2:** (**a**) The regression analysis result (**b**) Mean and variance along with F-test variance test result for conventional and HD montages in dorsal (marked in red) and ventral (marked in green) ROIs. (Level of significance denoted by * <0.05. ** <0.01, *** <0.001).

**(a)** Regression Analysis Results					
ROIs	R^2^	Montage		Montage × Patient Group	
		t-Value	*p*-Value	t-Value	*p*-Value
L_Inferior_Parietal_Lobule_ACD	0.205	−4.84	<2 × 10^−16^ ***	−1.62	0.000002 ***
L_Angular_Gyrus_ACD	0.162	−3.47	0.000628 ***	−1.06	0.045 *
L_MiddleTemporalGyrus_ACD	0.11	−2.76	0.0062 **	−1.71	0.08
L_InferiorTemporalGyrus_ACD	0.12	−2.63	0.0092 **	1.03	0.03 *
L_Inferior_Parietal_Lobule_Dose	0.197	4.01	0.000086 ***	−2.84	0.0048 **
L_Angular_Gyrus_Dose	0.163	4.23	0.000035 ***	−3.64	0.00034 ***
L_MiddleTemporalGyrus_Dose	0.09	−1.71	0.045 *	0.13	0.52
L_InferiorTemporalGyrus_Dose	0.10	1.01	0.031 *	−0.58	0.55
(b) Mean ± var of conventional and HD montages along with F-test variance test for each ROI					
ROIs		Conventional Mean ± std	HD Mean ± std	F-test variance (F, *p*), variance ratio	
L_Inferior_Parietal_Lobule_ACD		0.347 ± 0.03	0.182 ± 0.05	(F = 2.67, *p* < 0.001 ***), 2.76	
L_Angular_Gyrus_ACD		0.3 ± 0.24	0.19 ± 0.29	(F = 1.6, *p* = 0.01 **), 1.6	
L_MiddleTemporalGyrus_ACD		0.31 ± 0.023	0.21 ± 0.029	(F = 0.9, *p* = 0.001 **), 0.93	
L_InferiorTemporalGyrus_ACD		0.3 ± 0.034	0.19 ± 0.04	(F = 0.516, *p* = 0.7), 0.517	
L_Inferior_Parietal_Lobule_Dose		2.22 ± 6.27	2.51 ± 6.49	(F = 0.25, *p* < 0.001 ***), 0.252	
L_Angular_Gyrus_Dose		2.24 ± 3.95	2.60 ± 4.17	(F = 0.34, *p* < 0.001 ***), 0.35	
L_MiddleTemporalGyrus_Dose		2.24 ± 2.92	2.71 ± 5.25	(F = 0.29, *p* < 0.001 ***), 0.289	
L_InferiorTemporalGyrus_Dose		2.18 ± 3.64	2.50 ± 5.26	(F = 0.305, *p* < 0.001 ***), 0.305	

## Data Availability

The original data presented in one of the cohorts of the study (dementia) are openly available in LASI -DAD study https://lasi-dad.org/, accessed on 2 February 2024. The data presented in other two cohorts (depression and healthy volunteers) are available on request from the corresponding author due to restrictions in the consent obtained from the patients.

## Supplementary Materials

The following supporting information can be downloaded at: https://www.mdpi.com/article/10.3390/brainsci14121162/s1, Table S1: The detailed results of the regression analysis; Figure S1: DTDI for dorsal and ventral ROIs in dementia, depression and healthy showing no statistically significant difference.
