# Supplementary material for: Personalized Dose Selection for Treatment of Patients with Neuropsychiatric Disorders Using tDCS
Source: Brain Sci. 2024 Nov 21;14(12):1162. doi: 10.3390/brainsci14121162 (PMC11674973; doi:10.3390/brainsci14121162)
Supplement: Supplementary file 1 [file brainsci-14-01162-s001.zip › brainsci-3203500-supplementary.pdf]

SUPPLEMENTARY

Personalized Dose Selection for Treatment of Patients with Neuropsychiatric Disorders using tDCS

Sagarika Bhattacharjee[1*, MD, PhD], Rajan Kashyap[2*, PhD], Vanteemar S Sreeraj[3, MD], P. T. Sivakumar[3, MDΨ], Ganesan Venkatsubramanian[3, MD, PhD], John E. Desmond[6, PhDΨ], SH Annabel Chen[7,8,9, PhD], T N Sathyaprabha[1, MD, PhD.] & Kaviraja Udupa[1, MD, PhD]

Regression Analysis

The estimated marginal means and post hoc comparison of regression analysis with ACD, Personalised Dose and DTDI values at each target ROIs of the dorsal and ventral pathway as dependent factor and patient-group (dementia, depression, and healthy volunteer) and montage-type (conventional and high definition) as dependent factor.

Table S1: The detailed results of the regression analysis

DORSAL PATHWAY									
	**Avergae Current Density (ACD)**								
Left_Inferior _Parietal_Lobule	Montage	Patient_group	emmean	SE	estimate	SE	df	t.ratio	p.value
	Conventional	Dementia	0.352	0.026	0.1781	0.0368	194	4.84	<.0001***
	High_definition	Dementia	0.174	0.026					
	Conventional	Depression	0.279	0.0368	0.1294	0.052	194	2.487	0.133
	High_definition	Depression	0.149	0.0368					
	Conventional	Volunteer	0.409	0.0368	0.1849	0.052	194	3.553	0.0063**
	High_definition	Volunteer	0.224	0.0368					
Left_Angular_Gyrus	Montage	Patient_group	emmean	SE	estimate	SE	df	t.ratio	p.value
	Conventional	Dementia	0.293	0.0216	0.1061	0.0305	194	3.476	0.0081**
	High_definition	Dementia	0.187	0.0216					
	Conventional	Depression	0.253	0.0305	0.0999	0.0432	194	2.313	0.1937
	High_definition	Depression	0.153	0.0305					
	Conventional	Volunteer	0.369	0.0305	0.1205	0.0432	194	2.79	0.0634
	High_definition	Volunteer	0.248	0.0305					
	**Personalised Dose**								
Left_Inferior _Parietal_Lobule	Montage	Patient_group	emmean	SE	estimate	SE	df	t.ratio	p.value
	Conventional	Dementia	2.6	0.308	-1.746	0.435	194	-4.012	0.0012**
	High_definition	Dementia	4.35	0.308					
	Conventional	Depression	2.29	0.435	0.401	0.616	194	0.652	0.9868
	High_definition	Depression	1.89	0.435					
	Conventional	Volunteer	1.78	0.435	0.476	0.616	194	0.773	0.9718
	High_definition	Volunteer	1.3	0.435					
Left_Angular_Gyrus	Montage	Patient_group	emmean	SE	estimate	SE	df	t.ratio	p.value
	Conventional	Dementia	2.08	0.248	-1.4859	0.351	194	-4.234	0.0005***
	High_definition	Dementia	3.57	0.248					
	Conventional	Depression	2.77	0.351	0.7281	0.496	194	1.467	0.6856
	High_definition	Depression	2.05	0.351					
	Conventional	Volunteer	2.03	0.351	0.7904	0.496	194	1.593	0.6044
	High_definition	Volunteer	1.24	0.351					
	**DTDI**								
Left_Inferior _Parietal_Lobule	Montage	Patient_group	emmean	SE	estimate	SE	df	t.ratio	p.value
	Conventional	Dementia	0.734	0.0409	0.1444	0.0578	194	2.5	0.1291
	High_definition	Dementia	0.589	0.0409					
	Conventional	Depression	0.771	0.0578	0.1427	0.0817	194	1.746	0.5031
	High_definition	Depression	0.629	0.0578					
	Conventional	Volunteer	0.696	0.0578	0.1462	0.0817	194	1.789	0.475
	High_definition	Volunteer	0.55	0.0578					
Left_Angular_Gyrus	Montage	Patient_group	emmean	SE	estimate	SE	df	t.ratio	p.value
	Conventional	Dementia	0.644	0.0424	0.03562	0.06	194	0.594	0.9913
	High_definition	Dementia	0.608	0.0424					
	Conventional	Depression	0.667	0.06	0.07748	0.0848	194	0.914	0.9426
	High_definition	Depression	0.589	0.06					
	Conventional	Volunteer	0.621	0.06	-0.00624	0.0848	194	-0.074	1

VENTRAL PATHWAY									
	**Avergae Current Density (ACD)**								
Left_Middle_temporal_gyrus	Montage	Patient_gr	emmean	SE	estimate	SE	df	t.ratio	p.value
	Conventional	Dementia	0.313	0.0238	0.093	0.0336	194	3.765	0.0433*
	High_definition	Dementia	0.22	0.0238					
	Conventional	Depression	0.292	0.0336	0.0882	0.0476	194	1.854	0.4337
	High_definition	Depression	0.204	0.0336					
	Conventional	Volunteer	0.343	0.0336	0.1075	0.0476	194	2.26	0.2158
	High_definition	Volunteer	0.236	0.0336					
Left_Inferior_Temporal_Gyrus	Montage	Patient_gr	emmean	SE	estimate	SE	df	t.ratio	p.value
	Conventional	Dementia	0.288	0.0267	0.09927	0.0377	194	3.02	0.03*
	High_definition	Dementia	0.189	0.0267					
	Conventional	Depression	0.293	0.0377	0.08121	0.0534	194	1.521	0.651
	High_definition	Depression	0.212	0.0377					
	Conventional	Volunteer	0.336	0.0377	0.15415	0.0534	194	3.31	0.01*
	High_definition	Volunteer	0.182	0.0377					
	**Personalised Dose**								
Left_Middle_temporal_gyrus	Montage	Patient_gr	emmean	SE	estimate	SE	df	t.ratio	p.value
	Conventional	Dementia	2.66	0.258	-0.349	0.365	194	-0.956	0.931
	High_definition	Dementia	3.01	0.258					
	Conventional	Depression	1.89	0.365	-0.435	0.516	194	-0.843	0.9591
	High_definition	Depression	2.33	0.365					
	Conventional	Volunteer	1.76	0.365	-0.753	0.516	194	-1.459	0.6909
	High_definition	Volunteer	2.51	0.365					
Left_Inferior_Temporal_Gyrus	Montage	Patient_gr	emmean	SE	estimate	SE	df	t.ratio	p.value
	Conventional	Dementia	1.89	0.295	-0.422091	0.417	194	-1.013	0.9133
	High_definition	Dementia	2.31	0.295					
	Conventional	Depression	2.61	0.417	0.000793	0.589	194	0.001	1
	High_definition	Depression	2.61	0.417					
	Conventional	Volunteer	2.35	0.417	-0.459374	0.589	194	-0.779	0.9707
	High_definition	Volunteer	2.81	0.417					
	**DTDI**								
Left_Middle_temporal_gyrus	Montage	Patient_gr	emmean	SE	estimate	SE	df	t.ratio	p.value
	Conventional	Dementia	0.708	0.0379	0.04705	0.0535	194	0.879	0.9512
	High_definition	Dementia	0.661	0.0379					
	Conventional	Depression	0.737	0.0535	0.08002	0.0757	194	1.057	0.8977
	High_definition	Depression	0.657	0.0535					
	Conventional	Volunteer	0.679	0.0535	0.01408	0.0757	194	0.186	1
	High_definition	Volunteer	0.665	0.0535					
Left_Inferior_Temporal_Gyrus	Montage	Patient_gr	emmean	SE	estimate	SE	df	t.ratio	p.value
	Conventional	Dementia	0.662	0.0455	0.0728	0.0643	194	1.131	0.8679
	High_definition	Dementia	0.589	0.0455					
	Conventional	Depression	0.719	0.0643	0.1008	0.091	194	1.108	0.8778
	High_definition	Depression	0.618	0.0643					
	Conventional	Volunteer	0.605	0.0643	0.0448	0.091	194	0.492	0.9964

Dose Target Determination Index (DTDI) for dorsal and ventral ROIs

The DTDI analysis for the four (dorsal and ventral) ROIs at left inferior parietal lobule, left angular gyrus, left temporal gyrus and left middle temporal gyrus for the three groups (Dementia, Depression, and Healthy) across the conventional- and HD-tDCS settings.

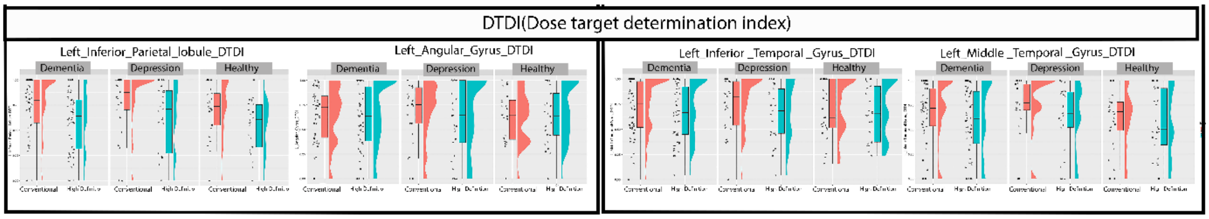

Figure S1: DTDI for dorsal and ventral ROIs in dementia, depression and healthy showing no statistically significant difference
